# Endothelial Cell GATA2 Modulates the Cardiomyocyte Stress Response through the Regulation of Two Long Non-Coding RNAs

**DOI:** 10.3390/biology11121736

**Published:** 2022-11-29

**Authors:** Natali Froese, Malgorzata Szaroszyk, Mortimer Korf-Klingebiel, Katrin Koch, Jan D. Schmitto, Robert Geffers, Denise Hilfiker-Kleiner, Christian Riehle, Kai C. Wollert, Johann Bauersachs, Joerg Heineke

**Affiliations:** 1 Medizinische Hochschule Hannover, Klinik für Kardiologie und Angiologie, 30625 Hannover, Germany; 2 Klinik für Herz-, Thorax-, Transplantations- und Gefäßchirurgie, 30625 Hannover, Germany; 3 Genomanalytik, Helmholtz-Zentrum für Infektionsforschung GmbH, 38124 Braunschweig, Germany; 4 Fachbereich Medizin–Der Dekan, Medicine, Philipps-Universität Marburg, Baldingerstraße, 35032 Marburg, Germany; 5 Department of Cardiovascular Physiology, Medizinische Fakultät Mannheim, European Center for Angioscience (ECAS), Universität Heidelberg, 68167 Heidelberg, Germany; 6 DZHK (German Center for Cardiovascular Research), Partner Site Heidelberg/Mannheim, 68167 Mannheim, Germany

**Keywords:** pressure overload, cardiac endothelial cells, paracrine signaling

## Abstract

**Simple Summary:**

Endothelial cells constitute the inner layer of blood vessels and they are the main constituent of the capillaries, which are the smallest blood vessels of the body that deliver nutrients and oxygen to the different organs. The heart contains an especially high number of capillaries in order to provide enough energy for the blood pumping activity of the cardiac muscle cells. While the contribution of these muscle cells for the development of heart failure has been investigated for decades, the role of endothelial cells in this deadly disease remains largely unclear. We showed in this study that elimination of the transcription factor GATA2 from endothelial cells leads to aggravated cardiac failure in mice with experimental transverse aortic constriction (TAC), which is a model for pressure overload of the heart as it occurs in longstanding arterial hypertension or narrowing of the aortic valve. We found that lack of GATA2 did not change the number of capillaries in the heart, but rather led to secretion of two so far unknown long non-coding RNAs (i.e. RNAs that do not code for proteins) from endothelial cells, which might trigger heart failure by acting on stress responsiveness of cardiac muscle cells.

**Abstract:**

Capillary endothelial cells modulate myocardial growth and function during pathological stress, but it is unknown how and whether this contributes to the development of heart failure. We found that the endothelial cell transcription factor GATA2 is downregulated in human failing myocardium. Endothelial GATA2 knock-out (G2-EC-KO) mice develop heart failure and defective myocardial signal transduction during pressure overload, indicating that the GATA2 downregulation is maladaptive. Heart failure and perturbed signaling in G2-EC-KO mice could be induced by strong upregulation of two unknown, endothelial cell-derived long non-coding (lnc) RNAs (AK037972, AK038629, termed here GADLOR1 and 2). Mechanistically, the GADLOR1/2 lncRNAs transfer from endothelial cells to cardiomyocytes, where they block stress-induced signalling. Thereby, lncRNAs can contribute to disease as paracrine effectors of signal transduction and therefore might serve as therapeutic targets in the future.

## 1. Introduction

Chronic heart failure is a common and deadly disease. In the Western world, it is mainly caused by myocardial infarction, chronic arterial hypertension, or abnormalities of the cardiac valves [1,2]. During these diseases, an increased workload is imposed onto the left ventricular chamber of the heart. As a consequence, the myocardium mounts a hypertrophic response with growth of individual cardiac myocytes in order to maintain the cardiac output. Although initially compensatory, pathological hypertrophy often leads to the development of heart failure in the long term [1,2]. Besides cardiomyocyte growth, there are also profound changes in the myocardial capillary network during disease progression: in the initial compensatory phase, angiogenesis and capillary density are increased [3,4,5], but in end-stage heart failure, capillary rarefaction is evident in the myocardium of mice and patients [6]. In addition to their crucial function for blood supply, capillary endothelial cells directly act on cardiomyocytes and regulate the growth and function of these cells. Co-culture experiments, for example, have demonstrated that endothelial cells direct the spatial organization of cardiomyocytes and also improve their survival, independent of perfusion [7]. In animal models, enhanced angiogenesis was associated with aggravated or unchanged cardiac hypertrophy as well as with improved or depressed cardiac function, depending largely on the means used to stimulate or inhibit angiogenesis [3,4,5,8,9]. Therefore, rather than the pure quantity of myocardial endothelial cells, their specific characteristics might be more important for their regulatory impact on cardiac structure and function. For example, the factors they release into the extracellular space might exert crucial regulatory effects on adjacent cardiomyocytes. A recent study revealed a highly irregular morphology of cardiac endothelial cells in heart failure, pointing towards a possible maladaptive role of the endothelium under these circumstances [10]. To date, only very few of the endothelial-derived factors with impact on cardiac myocyte growth or function are known; examples include neuregulin-1, endothelin-1, and CTRP9, which all induce cardiomyocyte hypertrophy [11,12]. Besides the secretion of proteins, it was recently demonstrated that endothelial cells can also shed small vesicles such as exosomes and thereby transfer non-coding RNAs to cardiac myocytes [13]. Even less is known about the responsible upstream regulators of EC-derived paracrine factors, and we hypothesized that select endothelial transcription factors might be altered in their expression during cardiac disease with consequences for paracrine signaling. GATA-binding protein 2 (GATA2) is known as a transcriptional regulator in endothelial cells that can be influenced in its activity by external mechanical stimulation by external mechanical stimulation, such as increased rigidity of the extracellular matrix [14]. GATA2 participates in the regulation of endothelial genes and ChIP sequencing revealed its DNA binding in the vicinity of genes important for endothelial identity [15]. Although siRNA-based downregulation of GATA2 in human umbilical vein endothelial cells as well as in mouse retinas strongly suggested an essential pro-angiogenic role; this was so far not found in genetic mouse models [16]. Systemic GATA2 knock-out mice die at embryonic day 10.5 because of severe anemia, but their vasculature appears morphologically normal at this stage [17]. Further studies using conditional *Gata2* inactivation or mutation of a cis-element in *Gata2* postponed embryonic lethality to embryonic day E16.5 or E14.5 and revealed lymphatic mispatterning or disturbances in the venous vascular cell integrity, respectively [17,18]. The latter was associated with profound changes in endothelial gene expression, supporting an important GATA2-dependent regulatory role in these cells, although this has not yet been addressed by genetic approaches in adult mice.

We demonstrate in this study that inducible genetic deletion of GATA2 in endothelial cells of adult mice precipitated cardiac failure during experimental pressure overload without impairment of myocardial capillarization. Instead, endothelial loss of GATA2 led to a strong upregulation of two related long non-coding RNAs in these cells, which are taken up by cardiac myocytes where they alter stress-dependent signal transduction and ultimately induce heart failure.

## 2. Materials and Methods

### 2.1. Animals

Tamoxifen-inducible endothelial cell-specific GATA2 knock-out mice were generated by crossing GATA2flox/flox mice [19] with Tie2ERT2Cre (in here: Tie2-CreER) transgenic mice or Cdh5 (PAC)-CreERT2 (in here: VE-Cadherin-CreER) mice. GATA2flox/flox and wild-type mice with and without Cre were used as controls. GATA2flox/flox x Tie2-CreER (G2-EC-KO) and control animals received tamoxifen (Sigma-Aldrich, Taufkirchen, Germany) in their diet (400 mg/kg food pellets for 6 weeks, i.e., 40 mg/kg body weight/day). GATA2flox/flox x VE-Cadherin-CreER (G2-EC-KO, VE-Cad) and controls were injected intraperitoneally with a dose of 0.5 mg/mouse/day of tamoxifen for 5 consecutive days. Administration of tamoxifen was started at the age of 5–6 weeks. Constriction of the transverse aortic arch (TAC) was performed in 8- to 12-week-old mice around a 27-gauge needle. Echocardiography was performed with a linear 30MHz transducer (Vevo770, Visualsonics, Amsterdam, Netherlands) in mice that were sedated with 1–1.5% isoflurane and placed on a heating pad to maintain body temperature. LV end-diastolic area (LVEDA), end-diastolic average wall thickness (Wth), and end-diastolic volume (EDV) as well as end-systolic area (LVESA) and endsystolic volume (ESV) were recorded or calculated from the short axis view. Ejection fraction was calculated as [(EDV-ESV)/EDV] × 100. For the assessment of mouse survival after TAC, mice were followed and inspected daily for 15 days. No mortality was observed in sham-operated mice. Mice that died before the end of the planned experimental period were excluded from the analysis.

At the end of the experiments, mice were weighed and then euthanized. Hearts and lungs were quickly removed from the thoracic cavity. After the removal of blood, the heart and lung weights were determined. Male and female animals were used in similar ratios in experimental groups that were compared to each other. All procedures involving the use and care of animals were performed according to the Guide for the Care and Use of Laboratory Animals published by the National Research Council (NIH Publication No. 85-23, revised 1996) and the German animal protection code. All animal procedures were approved by our local state authorities (the Lower Saxony State Office for Consumer Protection and Food Safety, Germany 33.9-42502-12-10/0016 and 33.19-42502-04-14/1403).

### 2.2. Human Tissue and Serum Samples

Studies on human heart tissue were permitted by the Massachusetts General Hospital Institutional Review Board (Boston, MA, USA), and by the Ethical Committee of the Hannover Medical School, Hannover, Germany (Az. Z 14.06-A 1871-30724/98). Transmural left-ventricular samples of the cardiac apex were obtained during implantation of an LVAD (Supplemental Data Table S1). The control human heart tissue was derived from victims of motor vehicle accidents, gunshot wounds, or from healthy heart organ donors when the organ was ineligible for transplantation.

### 2.3. Cell Culture

Primary juvenile cardiac endothelial cells were isolated from the hearts of 7–11-day-old mice as previously described [20]. In short, the mouse hearts were digested by collagenase type 1 (Worthington, Minnesota, USA) with subsequent purification of endothelial cells by Dynabeads (Invitrogen, ThermoFisher Scientific, Waltham, MA, USA) coated with CD31 antibodies (BD Pharmingen, Heidelberg, Germany). The cells were plated on gelatine-coated cell culture plates. After growing to confluency, the cells were purified again with Dynabeads coated with CD102 (BD Biosciences, Franklin Lakes, NJ, USA) antibodies and plated on gelatine-coated cell culture plates for experiments. Adult heart endothelial cells were isolated from the hearts of adult mice using CD146-coated micro beads with MACS technology from Miltenyi. The primary isolated ECs were >90% pure, as determined by FACS analysis after staining for CD31 [21]. The isolated cells were directly used for RNA extraction. Neonatal rat ventricular cardiomyocytes (NRCMs) were isolated from 1–3-day-old Sprague–Dawley rats by Percoll density gradient centrifugation as previously described [22]. On the day after isolation, the cells were switched to serum-free media. HEK293 cell line (Cell Line 293 ACC: 305, DSZM), NIH3T3 (ATCC), and C166 mouse embryonic yolk sac endothelial cells (C-166, ATCC) were cultured in DMEM containing 10% FCS.

### 2.4. Plasmids and Adenoviruses

To generate the luciferase reporter plasmids harboring the putative GADLOR1 and 2 promoter DNA, the sequences 3365 bp upstream of GADLOR1 and 3581 bp upstream of GADLOR2 were separately cloned into the pGL2 basic vector (Promega, Madison, WI, USA) to generate pGADLOR1-Luc and pGADLOR2-Luc. Transfections were performed using Lipofectamine 2000 reagent (Invitrogen, ThermoFisher Scientific, Waltham, MA, USA).

Ad.βgal (Ad.Control), Ad.Cre, Ad.GATA-luc, and Ad.NFAT-luc were previously described [23,24,25]. To generate GADLOR-expressing recombinant adenoviruses (Ad.GADLOR1 and 2), the mouse cDNAs of AK037972 (GADLOR1) and AK038629 (GADLOR2, both from Source BioScience) were subcloned into the pShuttleCMV vector and adenoviruses were generated using the AdEasy Adenoviral Vector System (Agilent Technologies, Santa Clara, CA, USA). For GATA2 overexpression, human GATA2 cDNA (BC006793, Open Biosystems) was subcloned into the pShuttleCMV vector, a myc-tag was introduced at the N-terminus (pshuttleCMV-GATA2-myc), and an adenovirus was generated. Adenoviral infection of cultured cells was conducted at 37 °C for 2 h. Subsequently, the cells were washed three times with PBS before cell culture media was applied.

### 2.5. Co-Culture of Endothelial Cells and Cardiomyocytes

Adenoviruses encoding for either β-galactosidase (Ad.Control) or Cre recombinase (Ad.Cre) were added to primary juvenile endothelial cells for two hours on the day after purification with CD102 antibodies. For co-culture, neonatal rat cardiomyocytes were plated on top of the endothelial cells 24 h later. Cell size was determined by planimetry after immunofluorescence staining for α-actinin (Sigma-Aldrich, Taufkirchen, Germany).

### 2.6. Stretch Assay

To produce cyclic stretch in vitro, a computerized Flexcell strain unit (FX-5000T, Flexcell International, Burlington, NC, USA) was employed. Cells were seeded on a Bioflex culture plate on type I collagen substrate (Dunn Labortechnik, Asbach, Germany). Twenty-four hours after infection with Ad.GATA-Luc, cells were subjected to a 15% radial stretch (1 Hz) for twenty-four hours. Controls consisted of cells seeded on the Bioflex culture plate without cyclic stretch.

### 2.7. Luciferase Assay

NIH3T3 cells were transiently transfected with pGADLOR1/2-Luc constructs and co-transfected with pshuttleCMV-GATA2-myc or empty plasmid.

NRCMs were infected with Ad.NFAT-Luc. Primary juvenile endothelial cells were infected with Ad.GATA-Luc and subjected to stretch. All cells were harvested 48 h after transfection/infection using Passive Lysis Buffer (Promega, Madison, WI, USA) and luciferase activity was measured. Values were normalized to total protein as described previously [21].

### 2.8. RNA Isolation and Real-Time PCR

RNA from cultured cardiomyocytes, mouse hearts, or human hearts was isolated with the Trifast reagent (Peqlab, Erlangen, Germany). For RNA isolation from endothelial cells, the NucleoSpin RNA II kit (Macherey Nagel, Düren, Germany) was used according to the manufacturer’s protocol.

cDNA was generated from RNA with the Maxima H Minus First Strand cDNA Synthesis Kit (Thermo Scientific, Waltham, MA, USA) using standard procedures. Quantitative PCR was performed using the Maxima SYBR Green qPCR Master Mix (Thermo Scientific, Waltham, MA, USA) following the kit directions with the MX4000 multiplex QPCR system from Stratagene. Gene expression was normalized to Gapdh mRNA expression. The value of control samples was set to 1. The following qPCR primers were used in this study: LNA long RNA GapmeR scramble control: 5′-AACACGTCTATACGC-3′; LNA long RNA GapmeR targeting GADLOR1: 5′-TGCAAGATGATTGAGA-3′LNA long RNA GapmeR targeting GADLOR2: 5′-TCAAGGATAGAGGTT-3′- all from Exiqon (500175); qPCR primers for mouse Gapdh: 5′- CCGCATCTTCTTGTGCAGT -3′ and 5′- CATCACCTGGCCTACAGGAT -3′; β-MHC: 5′- AGGCAAGGCAAAGAAAGGCTCATC -3′ and 5′- GCGTGGAGCGCAAGTTTGTCATAA -3′; α-MHC: 5′- ACTGTGGTGCCTCGTTCC -3′ and 5′- GCCTCTAGGCGTTCCTTCTC -3′; SERCA2a: 5′- ACGTGCCTGGTGGAGAAGATGAAT -3′ and 5′-ATCTTGCTCATGGATGTCCGGCTT -3′; Gata2: 5′- GCACCTGTTGTGCAAATTGT -3′ and 5′- AGGGCGGTGACTTCTCTTG -3; BNP: 5′- CTCAAGCTGCTTTGGGCACAAGAT -3′ and 5′- AGCCAGGAGGTCTTCCTACAACAA -3′; RCAN1.4: 5′- GCTTGACTGAGAGAGCGAGTC -3′ and 5′- CCACACAAGCAATCAGGGAGC -3′; GADLOR1: 5′- TTACATGGTTCCCTACCCAGACCA -3′ and 5′- GGGTGGCATGCAAGATGATTGAGA -3′; GADLOR2: 5′- AGGACTTGCAGGGACTCACA -3′ and 5′- TCAATAGCCATTCAGTTTTCAA -3′; mouse-specific GADLOR1: 5′- AGCTTGGGCAAACTCCTTA -3′ and 5′- GAAGTCTTAAAAATGCATGGC -3′; and mouse-specific GADLOR2: 5′- CACAGTGTGTCATATTTTGCA -3′ and 5′- CAAAGGAACACCTTCATAGC -3′.

### 2.9. Agilent Microarray

For gene expression profiling, the Mouse Gene Expression kit from Agilent was used. An amount of 200 ng of total RNA was transcribed into cDNA, amplified using T7 RNA polymerase while incorporating cyanine 3-labeled CTP, and then hybridized according to the manufacturer’s protocol (Quick Amp, Agilent, Santa Clara, CA, USA). Signal intensities were extracted from scan images using Feature Extraction Software v10.7.3.1. The raw data were deposited in the GEO expression database (http://www.ncbi.nlm.nih.gov/geo/) under the accession number GSE93596 accessed on 31 December 2017. Raw data were further analyzed using R package “Limma”. Raw data were log2 transformed and quantile normalized. For testing differential gene expression, normalized data sets were filtered for informative genes (showing at least expression values >log2(15) in more than two samples). Datasets were tested across all groups (ANOVA) or pairwise using linear models to assess differential expression in the context of the multifactorial designed experiment. For statistical analysis and assessing differential expression, Limma used an empirical Bayes method to moderate the standard errors of the estimated log-fold changes.

### 2.10. Immunoblot Analysis

Immunoblotting was performed using standard procedures with antibodies to the following proteins: phospho-Akt (9271), total-Akt (9272), phospho-p38 MAPK (9211), total-p38 MAPK (9212), phospho-ERK1/2 (9101), ERK 1/2 (9102), phospho-SAPK-JNK (9251), total-SAPK-JNK (9252), myc-tag (2276) (all from Cell Signaling Technology, Danvers, MA, USA), GATA2 (ab109241, Abcam), and actin (C6198, Sigma-Aldrich, Taufkirchen, Germany). Densitometry of protein bands was performed using QuantityOne (The Discovery Series Quantity One 1-D Analysis Software Version 4.6.6, PC, Bio-Rad) software.

### 2.11. ChIP Assay

The chromatin immunoprecipitation (ChIP) assay was performed according to manufacturer’s directions (ChIP Assay kit, Millipore, Burlington, MA, USA) on the cells overexpressing myc-tagged-GATA2 protein. Myc-tag antibodies (Cell Signaling, Danvers, MA, USA) and normal mouse IgG (Santa Cruz, Dallas, TX, USA) were used for immunoprecipitation. Sequences of the primer used to amplify regions of GADLOR promoter were: ChIP assay primers for GADLOR1: primer 1: 5′- CAATTACAAACACTGAAGTAACAATTT -3′ and 5′- GCCCTCTTCTGGCCTCTAAA -3′; primer 2: 5′- GGCTGAGCCATTTCATCTCT -3′ and 5′- TATCCACGTGCACTCACACA -3′; primer 3: 5′- ATTTGTTCGGTTTGGCAATG -3′ and 5′- GACGGCTCAAGAGGTAAGCTA -3′; primer 4: 5′- GAGACAGGCACCCAGAAGAC -3′ and 5′- CACACCCCTCTTTTGCTTTC -3′; and primer 5: 5′- CTCACCTCTTCCTGGCTCAC -3′ and 5′- TCCCTTTTCCATTCCTCTCA -3′.

ChIP assay primers for GADLOR2: primer 1 were: 5′- TTCCTTGCTGGGTATCTTGG -3′ and 5′- GCCCTCTTCTGGCCTCTAAA -3′; primer 2: 5′- CGTGGCAGCAAGTTAAATCA -3′ and 5′- CTGTGGCAGTGTTGCCTCTA -3′; primer 3: 5′- TTTGCATTTCTGATACTTACTGGA -3′ and 5′- CGAGGTCATTGAAATCGCTTA -3′; primer 4: 5′- GGAGACCGAGATCAAGCAAA -3′ and 5′- TGGTCCTTTGAACCCTCATT -3′; and primer 5: 5′- TTGAAGATGTTGCAAACAAGAA -3′ and 5′- AGGTGAAGTGGGATTTGTGC -3′.

### 2.12. Histological Analysis

Cryosections (7 µm in thickness) were stained with TRITC-conjugated wheat germ agglutinin (WGA, Sigma-Aldrich, Taufkirchen, Germany) to outline cardiomyocytes and with Isolectin B4 (Vector Laboratories, Newark, CA, USA) to visualize endothelial cells and capillaries. Fibrosis was detected with the Sirius Red staining method.

Immunofluorescence staining was performed using standard procedures. Antibodies used for immunofluorescence staining were: hosphor-p38 MAPK (#9211), hosphor-Akt #(9271) (both from Cell Signaling Technology, Danvers, MA, USA), α-actinin (A7811, Sigma-Aldrich, Taufkirchen, Germany) followed by Anti-Rabbit/Mouse IgG Alexa Fluor^®^ 488 (#4412), or Anti-Rabbit/Mouse IgG Alexa Fluor^®^ 555 secondary antibody (#4409, NEB, Ipswich, MA, USA). Nuclear staining was performed with Mounting Medium (#HP20.1, Roth, Karlsruhe, Germany) with DAPI.

### 2.13. Statistical Analysis

All values are presented as means ± sem. The sample size was chosen as a result of previous experience regarding data variability in similar models and experimental set-up. No statistical method was used to predetermine the sample size. All experiments were carried out in at least 3 biological replicates. The number of biological replicates (number of mice, samples, or cell culture dishes) is demonstrated in the Figures. Randomization was not used in this study. The investigators were blinded for mouse genotype and treatment during surgeries, echocardiography, organ weight determination, and all histological and immunofluorescence quantifications. The variance was comparable between groups and normality was assumed, when possible. Adequate tests were chosen to assess statistical significance. Multiple groups were compared by one-way repeated measures analysis of variance (ANOVA) followed by the Sidak´s multiple comparisons test or by Student’s t-test when comparing two experimental groups. Mouse mortality after TAC surgery was analyzed by the log-rank test. A two-tailed p value of less than 0.05 was considered significant. All statistics were calculated with the Graph Pad Prism 6 software.

## 3. Results

### 3.1. Reduced GATA Activation upon Mechanical Stretch in Endothelial Cells

**Figure 1:**
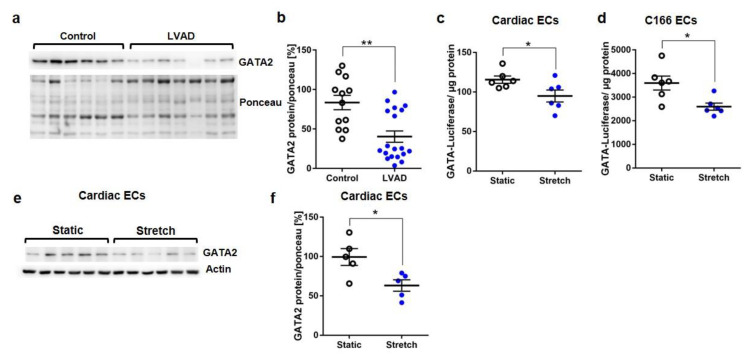
GATA2 is downregulated in endothelial cells upon mechanical stress. (**a**) Representative immunoblots and quantification for (**b**) GATA2 from control (healthy) and failing hearts of patients undergoing left ventricular assist device (LVAD) implantation. Ponceau staining was the loading control. Luciferase assay from primary cardiac endothelial cells (and **c**) C166 endothelial cells (ECs) (**d**) infected with Ad.GATA-Luc and subjected to mechanical stretch or not (static) for 24 h. (**e**) Immunoblot for GATA2 and quantification of (**f**) primary cardiac ECs treated for 24 h, as indicated. Data are mean ± sem; the number of biological replicates is shown in bars; * *p* < 0.05, ** *p* < 0.01.

GATA2 as a prominent endothelial transcription factor can be influenced by mechanical stimulation from the extracellular space. When we analyzed cardiac GATA2, which was previously shown to exist mainly in endothelial cells (and not in cardiomyocytes) in the heart, we found a strong downregulation of the GATA2 protein in cardiac specimens from patients undergoing implantation of a left ventricular assist device (LVAD, to support cardiac function in advanced heart failure) compared to healthy myocardium (Figure 1a,b, Table S1). Because heart failure is associated with a strong increase in mechanical overload, we hypothesized that this might be a crucial trigger for GATA2 dysregulation in this situation. In order to directly measure GATA factor activation in response to mechanical overload, we transduced primary cardiac mouse endothelial cells and C166 mouse endothelial cells with an adenovirus encoding for a GATA-dependent luciferase reporter construct and subsequently applied cyclic mechanical stretch (1 Hz, 15% elongation). Mechanical loading significantly reduced GATA-dependent transcriptional activity in both endothelial cell types (Figure 1c,d). Immunoblotting revealed a concomitant decrease in GATA2 protein levels in stretched versus static endothelial cells (Figure 1e,f).

### 3.2. Endothelial Specific GATA2 Knock-Out Induces Cardiac Failure upon Pressure Overload

**Figure 2:**
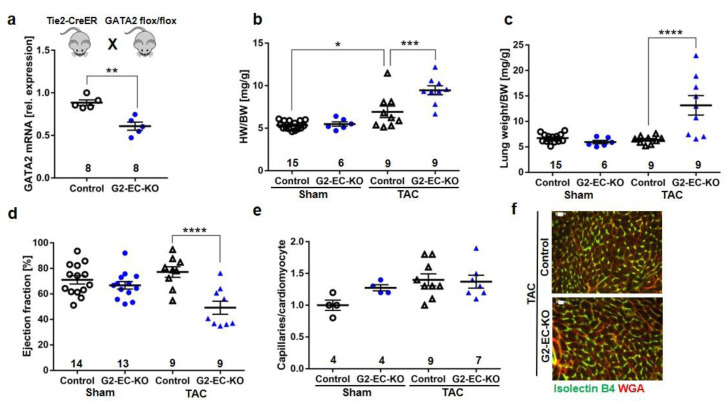
Endothelial GATA2 knock-out mice (Tie2-Cre-G2-EC-KO) develop heart failure during pressure overload (TAC). (**a**) qPCR of GATA2 mRNA in cardiac endothelial cells (EC) from indicated mice. (**b**) Heart weight/body weight (HW/BW), (**c**) lung weight/BW ratio, and (**d**) left ventricular ejection fraction (EF) 2 weeks after sham or TAC surgery. (**e**) Capillaries/cardiomyocyte ratio and (**f**) representative immunofluorescence pictures (scale bar, 20 µm). Data are mean ± sem; the number of biological replicates is shown in the graphs; * *p* < 0.05, ** *p* < 0.01, *** *p* < 0.001, **** *p* < 0.0001.

In order to assess the functional consequences of reduced endothelial GATA2 abundance during cardiac overload, we generated endothelial-specific GATA2 knock-out mice (G2-EC-KO) by breeding mice harboring a loxp-targeted GATA2 allele with endothelial Cre expressing Tie2-CreER mice (GATA2^flox/flox^ x Tie2-CreER, see Figure 2a). Endothelial cell-specific recombination was achieved in these mice by administration of tamoxifen containing chow for 6 weeks. Littermate GATA2^flox/flox^ mice as well as wild-type (WT) with and without Tie2-CreER fed with tamoxifen in the same manner were used as the control. Real-time PCR (qPCR) from isolated heart endothelial cells revealed a reduced *Gata2* mRNA abundance (by 50–60%) in G2-EC-KO mice (Figure 2a). To mimic pathological hemodynamic overload, experimental transverse aortic constriction (TAC) was performed on G2-EC-KO and control mice. G2-EC-KO mice exerted an increased heart weight/body weight ratio (HW/BW) and an increased lung weight/body weight ratio versus control mice as sign of enhanced cardiac growth and pulmonary congestion 2 weeks after TAC surgery, respectively (Figure 2b,c). In addition, echocardiography revealed a significantly diminished ejection fraction as an indicator of reduced left ventricular systolic function in G2-EC-KO mice after TAC (Figure 2d), but no changes were observed in left ventricular end-diastolic dimensions (LVEDA) or heart rate (Figure S1a,b).

The expression of parts of the embryonic gene program and the cardiomyocyte cross-sectional area was enhanced in G2-EC-KO mice after TAC (Figure S1c–g,i). No differences in heart or lung weights, cardiac function, or systemic blood pressure were noted between the groups of mice after sham surgery. Assessment of capillary density in the myocardium revealed no changes in the number of capillaries per cardiomyocyte between control or G2-EC-KO mice after TAC or sham surgery (Figure 2e,f). Similarly, cardiac tissue fibrosis was not changed due to reduced GATA2 levels in endothelial cells after TAC (Figure S1h).

**Figure 3:**
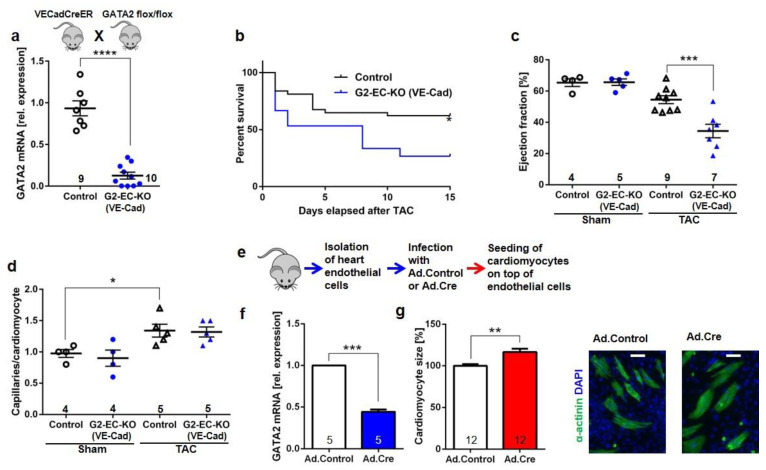
Endothelial GATA2 knock-out mice (VE-Cad-Cre-G2-EC-KO) develop heart failure and increased mortality during pressure overload (TAC). (**a**) qPCR of GATA2 mRNA in cardiac endothelial cells (EC) from indicated mice. (**b**) Kaplan–Meier survival curves after TAC (*n* = 37 control and *n* = 16 VE-Cad-G2-EC-KO) (**c**) EF of mice 4 weeks after TAC. (**d**) Capillaries/cardiomyocyte ratio. (**e**) Scheme of the experiment shown in (**f**), and (**f**) qPCR analysis of GATA2 mRNA in juvenile GATA2flox/flox ECs treated as indicated. (**g**) Quantification of the cell size of cardiomyocytes (CMs) grown on the top of ECs treated as shown. Representative immunofluorescence pictures stained as indicated (scale bar, 50 µm). Data are mean ± sem; the number of biological replicates is shown in the graphs; * *p* < 0.05, ** *p* < 0.01, *** *p* < 0.001, **** *p* < 0.0001.

In order to obtain a more efficient endothelial cell-specific removal of GATA2 in endothelial cells, we crossed GATA2^flox/flox^ mice with endothelial Cre-expressing VE-Cadherin-CreER mice. DNA recombination was induced by tamoxifen injections. The resulting G2-EC-KO (VE-Cad) mice exerted a significant reduction (by 90%) of *Gata2* mRNA expression in isolated cardiac endothelial cells compared to control mice (GATA2^flox/flox^, WT, and VE-Cadherin-CreER mice with tamoxifen injection, Figure 3a). Induction of pressure overload led to a significantly increased mortality in G2-EC-KO (VE-Cad) mice after TAC compared to control mice (Figure 3b). No mortality was observed after the sham operations. Echocardiography in surviving mice revealed a strongly reduced cardiac systolic function, but no changes in LVEDA or heart rate in VE-Cadherin-Cre-based G2-EC-KO mice compared to control mice after TAC surgery (Figure 3c and Figure S1j,k). The capillary/myocyte ratio was similarly increased in both groups of mice after TAC (Figure 3d) and no difference in myocardial fibrosis was noted (Figure S1l). Because these results suggested that endothelial cells with reduced GATA2 levels trigger the induction of cardiac hypertrophy and dysfunction, we established a co-culture system between mouse cardiac endothelial cells and rat cardiomyocytes to test whether a direct interaction between both cell types could be involved. Endothelial cells from hearts of GATA2^flox/flox^ mice were isolated and infected with an adenovirus coding for Cre-recombinase (Ad.Cre) or lacZ (Ad.Control) (Figure 3e). Ad.Cre infection led to reduced GATA2 levels in endothelial cells, and cardiomyocytes plated on top of these cells were more hypertrophied compared to those plated on Ad.Control-infected endothelial cells (Figure 3f,g).

### 3.3. Disturbed Cardiomyocyte Stress Signaling in Endothelial Specific GATA2 Knock-Out Mice

**Figure 4:**
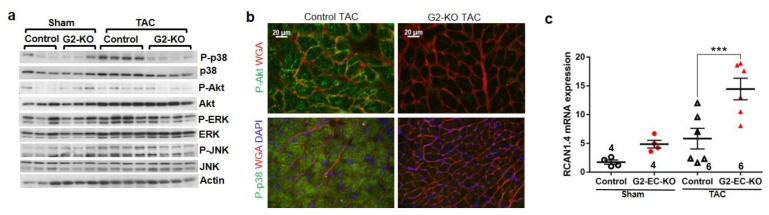
Regulation of cardiomyocyte signaling in endothelial GATA2 knock-out mice. (**a**) Immunoblots for indicated proteins of cardiac protein lysate 2 weeks after surgery. (**b**) Immuno-fluorescence of cardiac sections. (**c**) qPCR for RCAN1.4 mRNA from mouse hearts. Data are mean ± sem; the number of biological replicates is shown in the graphs; *** *p* < 0.001.

To further understand the molecular reasons for the development of cardiomyopathy in G2-EC-KO mice, we assessed pressure-overload-induced stress signaling by immunoblot (Figure 4a and Figure S2a–d). Compared to control mice, G2-EC-KO mice showed markedly reduced phosphorylation (i.e., activation) of p38MAPK as well as some degree of reduced Akt, ERK, and JNK phosphorylation after TAC. Immunofluorescence analysis with phospho-specific antibodies revealed that the lack of Akt and p38 activation in the knock-out mice occurred in cardiomyocytes (surrounded by WGA staining, Figure 4b). This was accompanied by increased cardiac RCAN1.4 mRNA expression in G2-EC-KO mice after TAC, which is indicative of increased calcineurin/NFAT activation [26] (Figure 4c).

### 3.4. The Long Non-Coding RNAs GADLOR1 and GADLOR2 Are Released in Response to Reduced GATA2 Expression and during Heart Failure

**Figure 5:**
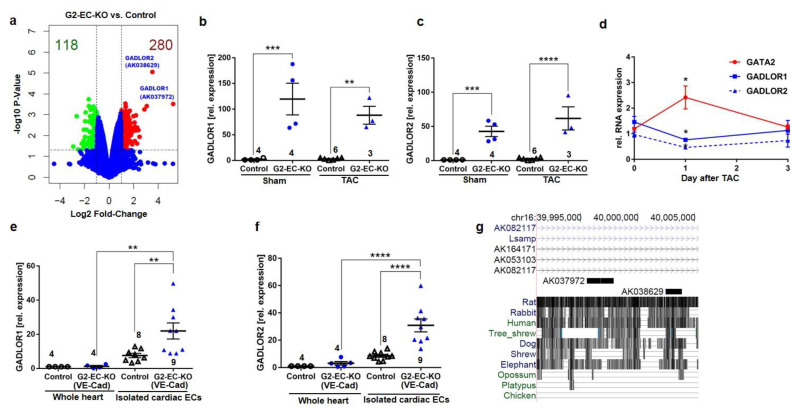
The lncRNAs GADLOR1 and 2 are upregulated in endothelial cells of G2-EC-KO mice. (**a**) Volcano plot of RNA expression from microarray of cardiac endothelial cells (EC) from indicated mice. Red dots indicate upregulated, green downregulated, and blue unregulated RNAs in G2-EC-KO ECs. (**b**,**c**) qPCR for GADLOR1 and 2 in mouse cardiac ECs. (**d**) qPCR of GATA2 and GADLOR1 and 2 in isolated EC; (**e**,**f**) qPCR of GADLOR1 and 2 in isolated cardiac ECs and whole hearts. (**g**) UCSC Genome Browser view of mouse chromosome 16, encoding GADLOR1 (AK037972) and 2 (AK038629). Data are mean ± sem; the number of biological replicates is shown in graphs; * *p* < 0.05, ** *p* < 0.01, *** *p* < 0.001, **** *p* < 0.0001.

Next, we wanted to assess how reduced GATA2 levels in endothelial cells might lead to cardiac failure. For this purpose, we isolated endothelial cells from the hearts of control and G2-EC-KO mice and analyzed global gene expression by microarray (Figure 5a). In total, 221 genes were significantly regulated by more than twofold (155 genes up and 66 genes were downregulated) in response to reduced GATA2 expression. As shown in Figure S3, gene ontology (GO, biological process, BP) showed upregulation of genes related to the cell cycle and mitosis and downregulation of genes related to the response to hypoxia, to cell movement, and to negative regulation of osteoclast differentiation (among others) in cardiac endothelial cells of G2-EC-KO versus control mice. We also performed a micro-array analysis in whole heart tissue of control and G2-EC-KO mice after TAC. GO-BP analysis from this data set (Figure S4) revealed the downregulation of circadian rhythms, negative regulation of cell growth, and response to cAMP-related genes, as well as the upregulation of genes, for example, related to neg. regulation of glucocorticoid receptor signaling, response to redox state, and response to insulin. 

The two most prominently upregulated genes in cardiac endothelial cells with reduced GATA2 (i.e., from G2-EC-KO mice) were unnamed RNAs with the accession numbers AK037972 (90-fold up versus control) and AK038629 (38-fold up versus control). Because their expression is dependent on GATA2, we named them **GA**TA**-D**ependent **Lo**ng non-coding **R**NA (GADLOR)1 and 2. We confirmed the strong upregulation of GADLOR1 and 2 RNA in cardiac endothelial cells isolated from G2-EC-KO mice versus control mice, but the exposure to TAC or sham surgery did not have an additional effect (Figure 5b-c). This upregulation of GADLOR1 and 2 indicated that GATA2 might suppress GADLOR expression. We therefore analyzed the expression of GATA2 and GADLOR1 and 2 in the endothelial cells of wild-type mice directly after TAC. We found that increased GATA2 expression is associated with decreased GADLOR expression in isolated endothelial cells in the acute phase of the cardiac stress response one day after TAC (Figure 5d). GATA2 deletion in G2-EC-KO (VE-Cad) mice also led to highly significant upregulation of GADLOR1 and 2 in cardiac endothelial cells, although to a somewhat lesser extent (Figure 5e–f). Comparison of GADLOR1 and 2 expression in purified cardiac endothelial cells versus heart tissue from control and G2-EC-KO (VE-Cad) mice revealed high enrichment of both RNAs in endothelial cells and relatively low GADLOR expression in the whole heart under baseline conditions (Figure 5e–f). GADLOR1 is 2296 base pairs (bp) long and analysis of possible open reading frames showed only scattered short fragments of possible protein coding regions, with a maximum length of 77 amino acids (Figure S5). None of these potential peptide fragments matched any known mouse peptides using blastp search (NCBI). GADLOR2 is 1393 bp long and, similar to GADLOR1, only scattered very short potential reading frames encoding either 35 or fewer amino acids were found, which do not exert any similarities to known mouse peptides (Figure S5). SDS-Gel and subsequent mass-spectrometry analysis did not identify any of the potential peptides in mouse C166 endothelial cells with adenoviral overexpression of GADLOR1 or GADLOR2. We therefore concluded that GADLOR1 and GADLOR2 are long non-coding (lnc) RNAs. Both are encoded on mouse chromosome 16 and are located in close proximity to each other, with only a 3601 bp linker in between them (Figure 5g). They are embedded in the intron after exon 1 of the *Lsamp* gene, which encodes the limbic system-associated membrane protein that is mainly expressed in the brain, where it regulates, for example, locomotion and anxiety. *Lsamp* gene expression was not detected in our micro-array from cardiac endothelial cells. Interestingly, the GADLOR1 and 2 sequences are highly conserved between mouse, rat, rabbits, humans, and dogs (Figure 5g).

**Figure 6:**
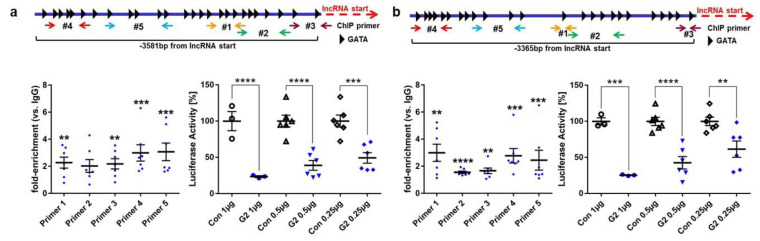
The putative promoter regions of GADLOR1 and 2 are bound and suppressed by GATA2. (**a**,**b**) Scheme of promoters of GADLOR1 and 2; potential GATA binding sites and primers for ChIP are shown. Below, left: Anti-myc ChIP for binding of myc-GATA2 to promoter regions of GADLOR1 (**a**) and 2 (**b**). Below, right: Luciferase activity after co-transfection with pGADLOR1-Luc or pGADLOR2-Luc and GATA2 or control plasmid. Data are mean ± sem; the number of biological replicates is shown in graphs; ** *p* < 0.01, *** *p* < 0.001, **** *p* < 0.0001.

We analyzed the sequence (−3365 bp) upstream of GADLOR1 as a potential promoter region (Figure 6a) and identified 24 possible GATA binding sites. A chromatin immunoprecipitation (ChIP) with anti-myc or control IgG from C166 mouse endothelial cells infected with a GATA2-myc-expressing adenovirus revealed GATA2 binding, especially in the region detected by the primer pairs #1 and #4, which also contain GATA consensus binding motifs (Figure 6a). In line with this, the GATA2 expression dose dependently suppressed luciferase activity when it was driven by the putative −3365 bp promoter region. Similar results were obtained when we analyzed the −3581 bp region upstream of GADLOR2, in which we identified 26 potential GATA binding sites: a ChIP assay demonstrated binding of GATA2 (to the region detected by primer pairs #4 and #5, both containing GATA consensus sites) and GATA2 potently suppressed the activity of the promoter in a dose-dependent manner (Figure 6b).

### 3.5. Disturbed Cardiomyocyte Stress Signaling due to GADLOR Uptake

**Figure 7:**
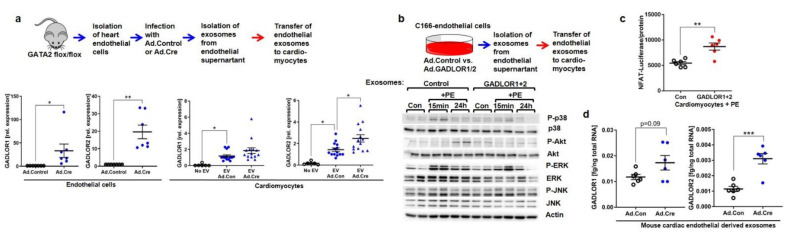
Regulation of cardiomyocyte signaling by endothelial GADLOR1 and 2. (**a**) Experimental scheme (**above**) and qPCR (**below**) of GADLOR1 and 2 in isolated GATA2flox/flox endothelial cells (ECs) treated with Ad.Cre or Ad.Control and in cardiomyocytes (CM) treated with exosomes from supernatants of these ECs. (**b**) Experimental scheme (**above**) and (**below**) immunoblots of indicated proteins from CMs treated as shown. (**c**) Luciferase activity from CMs infected with Ad.NFAT-Luc, treated with PE and exosomes. (**d**) qPCR of GADLOR1 and 2 in exosomes isolated from the supernatant of cardiac endothelial cells treated as shown. Data are mean ± sem; the number of biological replicates is shown in the graphs; * *p* < 0.05, ** *p* < 0.01, *** *p* < 0.001.

We hypothesized that the high expression of GADLOR in endothelial cells of the GATA2 mutant mice might trigger the dysregulation of signaling in cardiomyocytes. For this to be the case, GADLOR1 and 2 would have to be secreted by cardiac endothelial cells and taken up by cardiomyocytes to regulate signaling in these cells. To address this, we turned back to our in vitro system of cardiac endothelial cells from GATA2^flox/flox^ mice, in which we eliminated GATA2 by adenoviral Cre overexpression. Cre-expressing endothelial cells exerted increased levels of GADLOR1/2 (Figure 7a). Because RNA was reported to travel from one cell to another, for example, via extracellular vesicles such as exosomes, we isolated these from the supernatant of the Ad.Control- or Ad.Cre-infected mouse cardiac endothelial cells and transferred them to rat cardiomyocytes. Compared to rat cardiomyocytes without any exosome treatment, the transfer of exosomes from Ad.Control mouse endothelial cells led to strong enrichment of mouse GADLOR1/2 (as revealed by mouse-specific qPCR), indicating a constitutive release of GADLOR1 and 2 into endothelial cell-derived vesicles (Figure 7a). Cardiomyocyte enrichment of mouse endothelial GADLORs was significantly higher when exosomes from GATA2-depleted (i.e., Ad.Cre-infected) endothelial cells were applied. Because these results indicated transfer of GADLOR1/2 from cardiac endothelial cells to cardiomyocytes, we next wanted to assess the functional consequences of GADLOR uptake in cardiomyocytes. We established another model system, in which we overexpressed GADLOR1 and 2 by an adenoviral (Ad.) approach (Ad.GADLOR1 + Ad.GADLOR2), or lacz (Ad.Control) in mouse C166 endothelial cells, isolated exosomes from the supernatant of these cells, and transferred the exosomes to cardiomyocytes (Figure 7b). Because we had identified reduced activation of p38 and Akt kinases in G2-EC-KO hearts, we next tested the impact of GADLOR on cellular signal transduction. For this purpose, we stimulated the GADLOR or control exosome-treated cardiomyocytes with the α-adrenergic hypertrophy-inducing agonist phenylephrine (PE) for 15 min and 24 h. Assessment of kinase phosphorylation by immunoblotting demonstrated enhanced activation of p38, ERK, and JNK mainly 15 min and of Akt 24 h after the addition of PE to cardiomyocytes treated with control exosome, while in contrast, p38 as well as Akt activation were markedly blunted in cardiomyocytes treated with GADLOR1- and 2-containing exosomes (Figure 7b). ERK and JNK activation appeared unchanged. All blots shown in Figure 7b were also quantified by densitometry (Figure S6a–d). These results indicated GADLOR-dependent interference with adrenergic signal transduction. We also measured NFAT-dependent luciferase activity and found that it increased in PE-stimulated cardiomyocytes in response to GADLOR exposition (Figure 7c). Quantitative analysis of GADLOR1 and 2 expressions in isolated endothelial cell-derived exosomes revealed increased expression of both GADLORs (only a trend was observed for GADLOR1) in exosomes collected from GATA2-depleted endothelial cells (Figure 7d). In conclusion, we observed uptake of endothelial-derived GADLOR1 and 2 in cardiomyocytes and found exosomal GADLOR to interfere with p38 and Akt and to enhance NFAT transcriptional activation in response to PE, a pattern that we similarly observed in vivo in G2-EC-KO mice after TAC.

## 4. Discussion

Our study revealed the following: (1) During mechanical overload, the abundance of the transcription factor GATA2 is reduced in endothelial cells and GATA transcriptional activity in these cells is diminished. (2) Endothelial cell-specific depletion of GATA2 aggravates heart failure with perturbed cardiomyocyte stress signaling in response to experimental pressure overload in mice, but it is not associated with increased cardiac fibrosis or reduced capillary density. (3) Transcriptional profiling of cardiac endothelial cells with GATA2 depletion revealed a strongly induced expression of two related long non-coding RNAs, which we named GADLOR1 and GADLOR2. GADLOR1/2 are efficiently taken up by cardiomyocytes, where they consequently inhibit activation of p38 and Akt and lead to increased calcineurin/NFAT activity.

GATA2 became downregulated by mechanical overload in endothelial cells, and this promoted the development of heart failure, indicating that GATA2 is protective and prevents the expression of a maladaptive endothelial gene program. We confirmed a direct effect of endothelial cells on adjacent cardiomyocytes by employing an in vitro co-culture model of both cell types, in which endothelial GATA2 depletion induced enhanced cardiac myocyte hypertrophy. Endothelial GATA2 elimination was achieved in this study by using two different endothelial Cre driver lines, whereby the VE-Cadherin-CreER line is known to exert a higher specificity towards endothelial cells as well as a higher efficiency compared to the Tie2-CreER line [27,28]. Accordingly, we observed a stronger elimination of endothelial GATA2 in G2-EC-KO (VE-Cadherin) as well as a stronger phenotype with higher mortality of the mice after TAC.

GATA2 depletion resulted in a strongly increased expression of GADLOR1 and GADLOR2, which are two long non-coding RNAs encoded on chromosome 16 in close proximity to each other. At least in cardiac endothelial cells, GADLOR1 and GADLOR2 are not part of the same transcript (data not shown), but rather controlled by separate 5′ promoter regions, which are both bound and dose-dependently suppressed by GATA2. Indeed, lncRNAs expression was shown to be regulated by the same transcription factor binding sites compared to protein-coding genes, although some transcription factors were more prone to regulate lncRNAs expression and one of them was reported to be GATA2 [29].

GADLOR1 and 2 are likely secreted from cardiac endothelial cells within extracellular vesicles (like exosomes) and are efficiently taken up by cardiomyocytes. Similarly, the transfer of lncRNAs (TUC339 and linc-ROR) within extracellular vesicles was described among human hepatocellular cancer cells and might contribute to disease progression and resistance to chemotherapy [30,31]. Cardiomyocytes appear to be a prime target of extracellular vesicles, as the transfer of endothelial miR146a or miR21 * from fibroblasts to cardiomyocytes within vesicles has been described [13,32]. Since both GADLORs were induced in the GATA2 knock-out mice, we performed most of our experiments with the combination of GADLOR1 and 2. Indeed, the combination of both lncRNAs was more potent to influence signaling in cardiomyocytes and to induce heart failure compared to the administration of either lncRNA alone. How both GADLORs act together and whether they form dimers will have to be the subject of future studies. Protein binding by lncRNAs is commonly part of their mechanism of action [33], for instance, during recruitment of epigenetic factors to certain DNA regions [34]. It was only recently demonstrated, however, that lncRNAs also bind cytosolic signaling molecules and thereby modulate signal transduction [35,36].

Here, in a broader scheme, we have uncovered an exemplary mechanism by which capillary endothelial cells are able to attenuate intracellular stress signaling within a given tissue through a paracrine lncRNA-dependent mechanism. In this regard, we show that lncRNAs can interfere with signal transduction in surrounding cells, perhaps to synchronize the signaling response in a given tissue. Therefore, although modulation of cardiomyocyte stress-signaling by endothelial GADLORs is desirable to a certain extent, aggravated GADLOR abundance clearly triggers heart failure, which is at least in part the consequence of reduced p38 and Akt as well as enhanced calcineurin activation; cardiomyocyte-specific genetic depletion of p38 or general depletion of Akt1 in mice induced aggravated cardiac dysfunction after TAC [37,38]. p38 has been identified as a negative regulator of calcineurin/NFAT signaling in the myocardium [39]; therefore, the enhanced activation of myocardial calcineurin in G2-EC-KO and in the GADLOR-treated cells is likely the consequence of diminished p38 activation. Activated calcineurin/NFAT signaling, in turn, has also been shown to induce heart failure [40]. Decreased abundance of GATA2 in human failing hearts indicates that our findings might be relevant for patients with heart failure. Therapeutic strategies could be developed to reduce GADLOR expression and halt the progression of the disease in patients. Our endothelial GATA2 mutant mice are a useful model to develop these strategies.

Limitations of this study: Although we found increased GADLOR levels in mice with endothelial GATA2 depletion, we cannot infer that this is contributing to cardiac dysfunction in these mice. Although we showed dysfunctional stress-signaling and increased maladaptive calcineurin/NFAT activation due to the transfer of GADLOR-containing exosomes in vitro in cell culture, the effects of GADLORs in mice in vivo needs to be studied in future studies.

## 5. Patents

The following patent was granted: LncRNAs GADLOR1 and 2 for use in treating and preventing cardiac remodeling. US11208656 (granted 28 January 2021).

## Data Availability

The micro-array data that support the findings of this study have been deposited in the Gene Expression Omnibus (GEO) repository with the accession code GSE93596. The data can be accessed using the following link: https://www.ncbi.nlm.nih.gov/geo/query/acc.cgi?tkentken=wvsnakcalpkpxyl&acc=GSE9359GSE9359 (accessed on 31 December 2017). All other data are available from the corresponding author(s) upon reasonable request.

## Supplementary Materials

The following supporting information can be downloaded at: https://www.mdpi.com/article/10.3390/biology11121736/s1, Table S1: Clinical characteristics of the patients undergoing implantation of a left ventricular assist device (LVAD); Figure S1: Further characterization of endothelial GATA2 knock-out mice (G2-EC-KO); Figure S2: Signal transduction in GATA2 mutant mice; Figure S3: Micro-array analysis of endothelial gene-expression in cardiac endothelial cells of control and G2-EC-KO mice after TAC; Figure S4: Micro-array analysis of whole hearts of control and G2-EC-KO mice after TAC; Figure S5: Short open reading frames in the GADLORs; Figure S6: Signal transduction in cardiomyocytes during GADLOR exposure.
