# Supplementary material for: Endothelial Cell GATA2 Modulates the Cardiomyocyte Stress Response through the Regulation of Two Long Non-Coding RNAs
Source: Biology (Basel). 2022 Nov 29;11(12):1736. doi: 10.3390/biology11121736 (PMC9775420; doi:10.3390/biology11121736)
Supplement: Supplementary file 1 [file biology-11-01736-s001.zip › biology-1955560-supplementary.pdf]

Supplementary Table S1:
Clinical characteristics of the patients undergoing implantation of a left ventricular assist device (LVAD).
Data are displayed as mean ± SEM or in % of all patients as indicated. NYHA indicates New York Heart Association Class.

	**LVAD N=17**	**Normal values**
**Age (years)**	59.7±10.9	
**Sex (M/F, %)**	94.7/5.3	
**Echocardiography**		
**Ejection fraction (%)**	18.9±4.9	**>55**
**Septum thickness (mm)**	29.4±34.8	**≤11**
**Ischemic cardiomyopathy/ Dilated cardiomyopathy (%)**	35/65	
**Left ventricular end-diastolic diameter (mm)**	68±10	**33-56**
**Serum**		
**Creatinine (µmol/l)**	142.6±94	**59-104**
**Clinical Classification**		
**NYHA I [% of all patients]**	0	
**NYHA II [% of all patients]**	0	
**NYHA III [% of all patients]**	35.7	
**NYHA IV [% of all patients]**	**64.3**	

Supplemental Figure S1

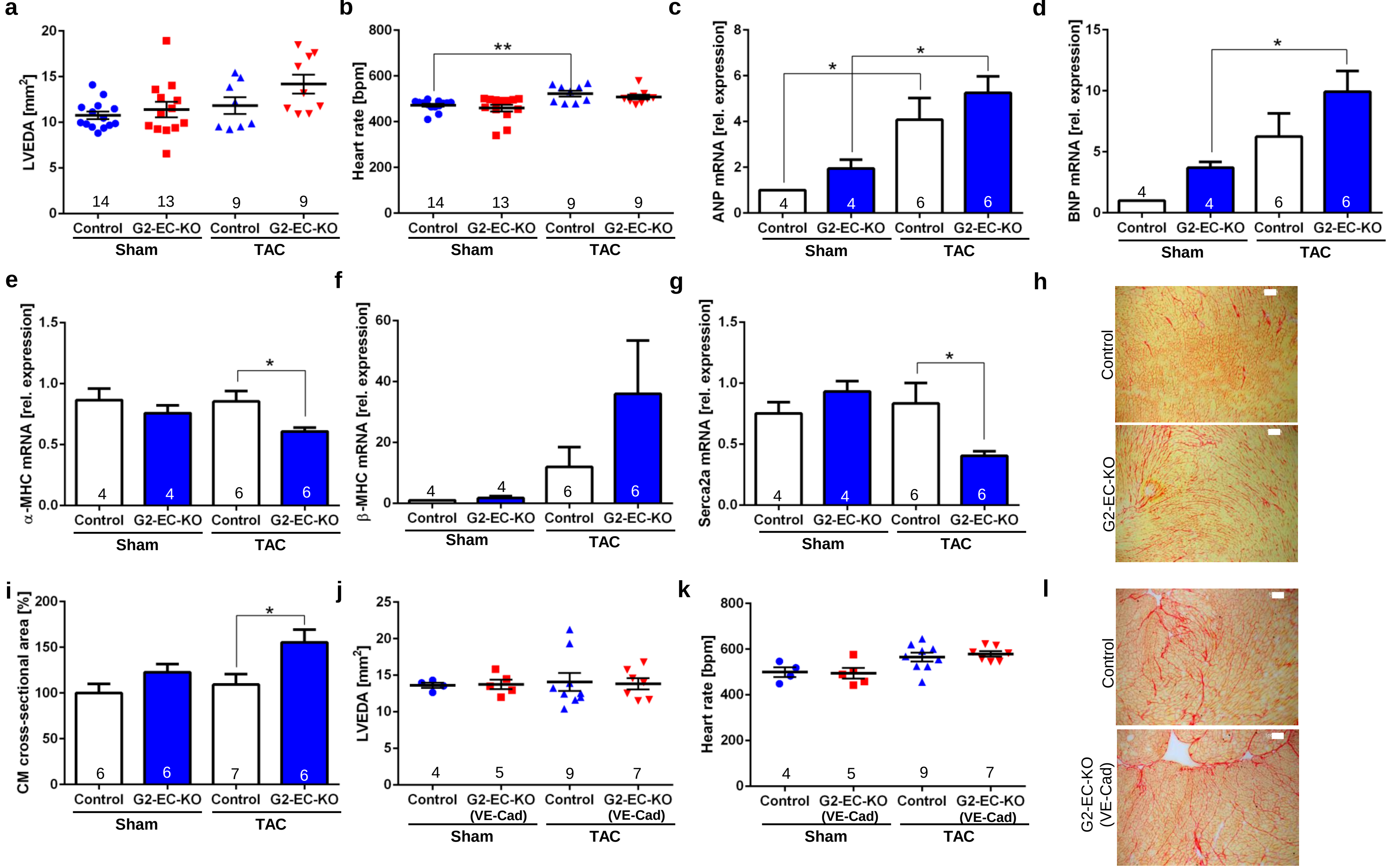

Supplemental Figure S1: Further characterization of endothelial GATA2 knock-out mice (G2-EC-KO). a, G2-EC-KO and control mice were subjected to transverse aortic constriction (TAC) or sham surgery and monitored for 2 weeks. Left ventricular end-diastolic area (LVEDA) and heart rate (**b**) were determined during echocardiography. **c-g**, qPCR analysis of ANP, BNP, α-MHC, β-MHC and SERCA2a mRNA expression from mouse hearts. **h**, Sirius-red staining (scale bar, 100µm) of heart tissue slides and quantification of cardiomyocytes cross-sectional area (**i**) of the indicated mice two weeks after TAC. **j-l**, G2-EC-KO (VE-Cad) and control mice were subjected to transverse aortic constriction (TAC) or sham surgery and monitored for 4 weeks. LVEDA (**j**) and heart rate (**k**) were determined. **l**, Sirius-red staining of heart sections (scale bar, 100µm). Data are mean±sem; the number of biological replicates is indicated within graphs; *p<0.05, **p<0.01.

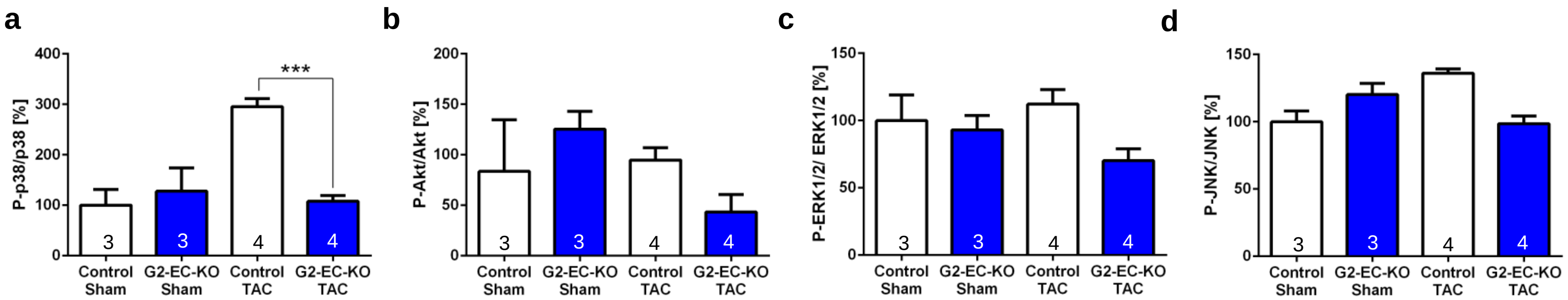

Supplementary Figure S2: Signal transduction in GATA2 mutant mice. a-d, Quantification of the immunoblots shown in the Figure 4a**.** Data are mean ± sem; the number of biological replicates is indicated within graphs; $*p<0.05$, $***p<0.001$.

Supplementary Figure S3

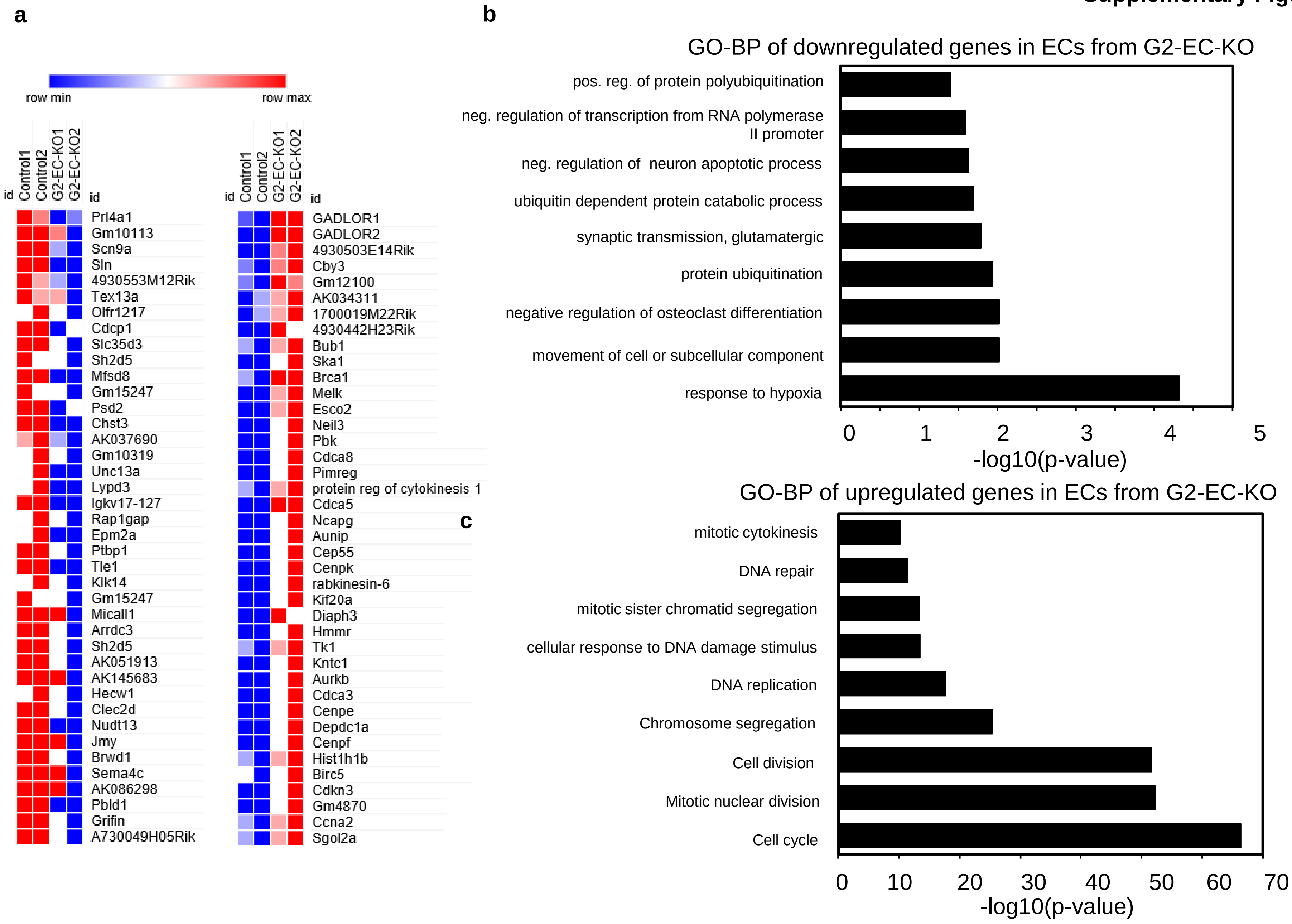

Supplementary Figure S3: Micro-array analysis of endothelial gene-expression in cardiac endothelial cells of control and G2-EC-KO mice after TAC. a, top 40 down- and up-regulated Genes between both genotypes. **b**, Gene ontology analysis, biological process (GO-BP) of down-regulated and of upregulated genes (**c**).

Supplementary Figure S4

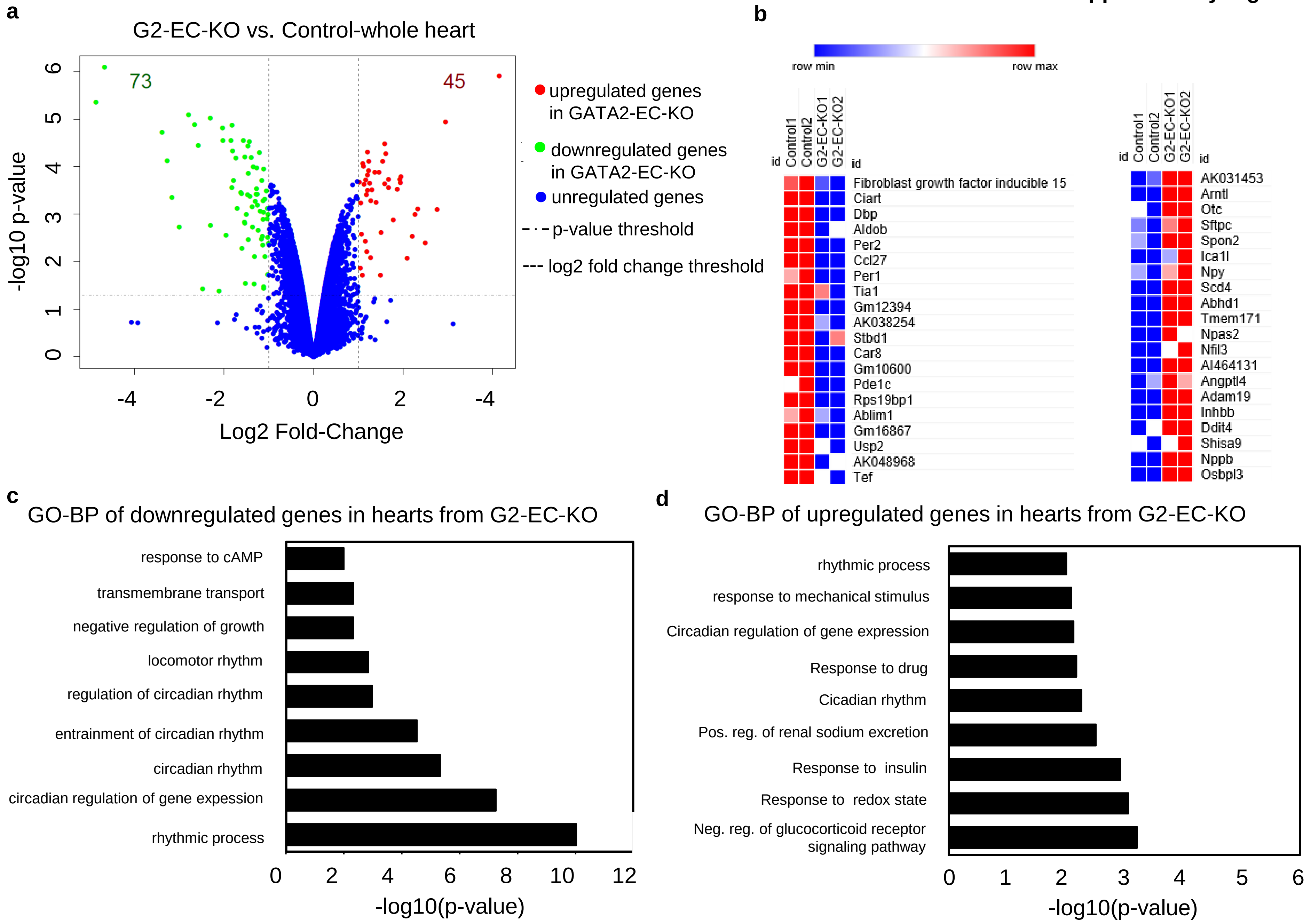

Supplementary Figure S4: Micro-array analysis of whole hearts of control and G2-EC-KO mice after TAC. a, volcano plot**. b,** top 20 down- and up-regulated genes between both genotypes. **c**, Gene ontology analysis, biological process (GO-BP) of down-regulated and of upregulated genes (**d**).

Supplemental Figure S5

GADLOR1

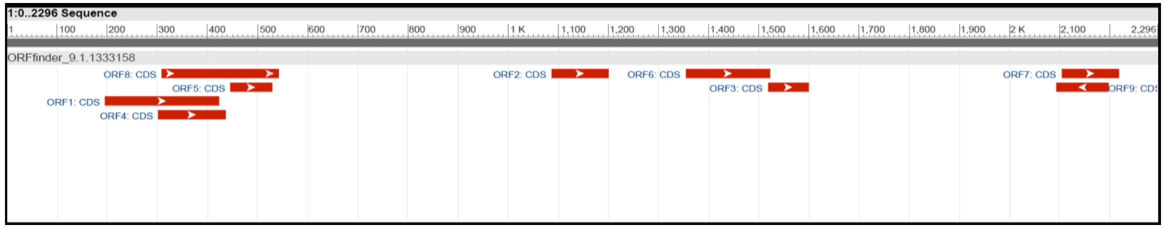

Label	Strand	Frame	Start	Stop	Length (bp | aa)
ORF8	+	3	309	542	234 | 77
ORF1	+	1	196	423	228 | 75
ORF6	+	2	1355	1522	168 | 55
ORF4	+	2	302	436	135 | 44
ORF2	+	1	1087	1200	114 | 37
ORF7	+	2	2105	2218	114 | 37
ORF9	-	3	2198	2094	105 | 34
ORF5	+	2	446	529	84 | 27
ORF3	+	1	1519	1599	81 | 26

GADLOR2

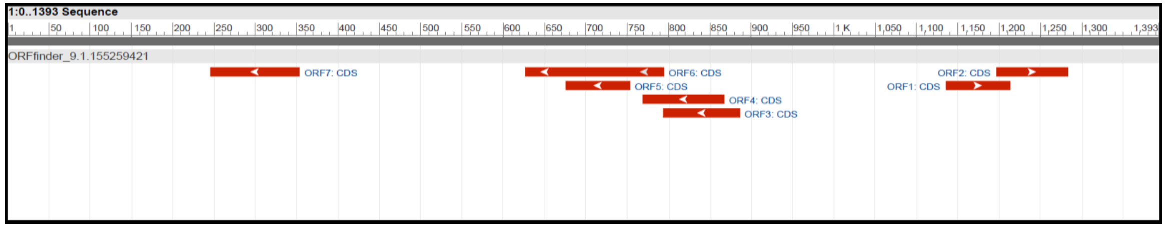

Label	Strand	Frame	Start	Stop	Length (bp | aa)
ORF6	-	3	794	627	168 | 55
ORF7	-	3	353	246	108 | 35
ORF4	-	2	867	769	99 | 32
ORF3	-	1	886	794	93 | 30
ORF2	+	3	1197	1283	87 | 28
ORF1	+	2	1136	1213	78 | 25
ORF5	-	2	753	676	78 | 25

Supplemental Figure S5: Short open reading frames in the GADLORs. Analysis of possible open reading frames (ORF) of GADLOR1 and GADLOR2; bp denotes base pairs, aa denotes amino acids.

Supplementary Figure S6

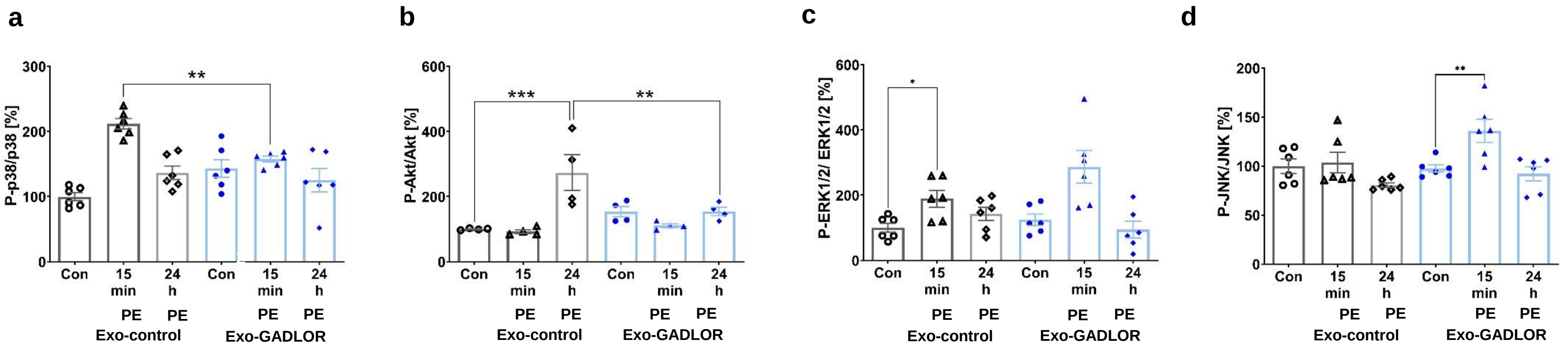

Supplementary Figure S6: Signal transduction in cardiomyocytes during GADLOR exposure. a-d, Quantification of the immunoblots shown in the Figure 7b and two additional experiments**.** Data are mean ± sem; the number of biological replicates is indicated within graphs; *p<0.05, **p<0.01, ***p<0.001.
